# A Fatal Case of Takotsubo Cardiomyopathy Secondary to Refractory Hypoglycemia in Severe Starvation: An Autopsy Case Report

**DOI:** 10.7759/cureus.23287

**Published:** 2022-03-18

**Authors:** Jin Kirigaya, Noriaki Iwahashi, Reiko Tanaka, Yoshiaki Inayama, Ichiro Takeuchi

**Affiliations:** 1 Advanced Critical Care and Emergency Center, Yokohama City University Medical Center, Yokohama, JPN; 2 Division of Cardiology, Yokohama City University Medical Center, Yokohama, JPN; 3 Department of Diagnostic Pathology, Yokohama City University Medical Center, Yokohama, JPN

**Keywords:** refeeding syndrome, venoarterial extracorporeal membrane oxygenation (va-ecmo), starvation, refractory hypoglycemia, takotsubo cardiomyopathy

## Abstract

A 56-year-old, severely malnourished man presented with loss of consciousness due to hypoglycemia. Echocardiography revealed left ventricular apical ballooning, indicating takotsubo cardiomyopathy. Although his caloric intake was gradually increased to avoid refeeding syndrome, hypoglycemia was refractory, and repetitive glucose administration was required. On day 4 of admission, he developed severe refractory hypoglycemia with a progressive decrease in blood pressure. Consequently, pulseless ventricular tachycardia followed by pulseless electrical activity developed. Although venoarterial extracorporeal membrane oxygenation was introduced, the patient did not respond to the treatment and died. Autopsy revealed myocardial degeneration and contraction-band necrosis, indicative of takotsubo cardiomyopathy. No coronary stenosis was observed. The liver showed moderate hepatocyte atrophy and autophagosomes, consistent with starvation and not with refeeding syndrome. We speculated that refractory hypoglycemia induced extreme catecholamine secretion, which led to severe complications of takotsubo cardiomyopathy, such as fatal arrhythmia and extremely low cardiac output. Early recognition of these critically ill patients and timely therapeutic interventions, including strict glycemic control and adequate caloric intake, may improve patient outcomes.

## Introduction

Takotsubo cardiomyopathy (TC) is characterized by a transient left ventricular (LV) regional wall motion abnormalities without relevant obstructive coronary artery disease, often resulting from stressful, emotional or physical triggering events. The precise pathophysiological mechanisms of TC are incompletely understood. However, there is considerable evidence that sympathetic stimulation is central to its pathogenesis [1]. TC has long been considered a benign disorder [2]. However, in recent years, it has been uncovered that TC can be associated with different complications including cardiogenic shock and life-threatening arrhythmias [1]. Although cardiac arrest is a relatively rare complication of TC (5.3%), it has been reported to be a poor prognostic factor [3]. 

Starvation is an important risk factor for hypoglycemia, the clinical course of which is still poorly understood, because severe repetitive and refractory hypoglycemia is uncommon. Several reports demonstrated that refractory hypoglycemia secondary to severe starvation induces cardiac complications, such as heart failure, fatal ventricular tachyarrhythmia, and TC [4-6]. However, the mechanism and precise treatment of these complications are unclear. Here, we report a case of TC secondary to hypoglycemia and starvation, whose pathogenesis and cause of death were investigated by autopsy.

## Case presentation

**Figure 1:**
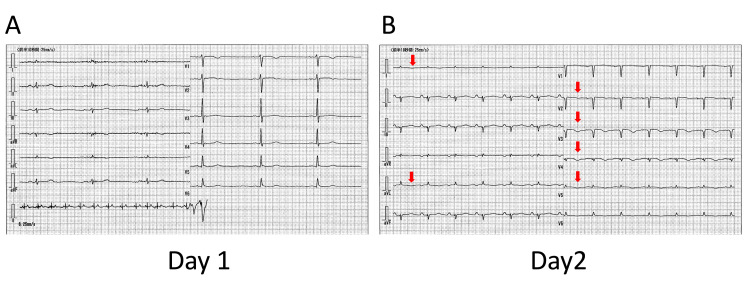
Electrocardiographic findings on admission (day 1) and day 2 ECG shows sinus bradycardia and no ST-segment changes on admission (A). On day 2, ECG shows negative T waves in I, aVL, and precordial leads from V2 to V5 (red arrows) (B). ECG, electrocardiogram.

A 56-year-old man with a medical history of schizophrenia was referred to our hospital with impaired consciousness due to severe hypoglycemia (<20 mg/dL) and hypothermia (29.4℃). After glucose infusion and rewarming, the patient’s consciousness gradually improved. Examination revealed the following: height 172 cm, weight 36 kg, body mass index 12.4 kg/m^2^, blood pressure of 138/94 mmHg, heart rate of 39 bpm, respiratory rate of 16 per minute, oxygen saturation of 100% on room air. He had no edema and dry skin. Pulmonary, abdominal, and neurological examinations were initially unremarkable. Blood test on admission showed the following: sodium, 134 mEq/L; potassium, 3.9 mEq/L; aspartate aminotransferase (AST), 870 IU/L; alanine aminotransferase (ALT), 682 IU/L; alkaline phosphatase, 589 U/L; γ-glutamyl transpeptidase, 88 IU/L; total protein, 4.1 g/dL; albumin, 2.8 g/dL; and brain natrium peptide, 332.3 pg/mL. Electrocardiography (ECG) showed sinus bradycardia and no significant ST change (Figure 1A).

**Figure 2:**
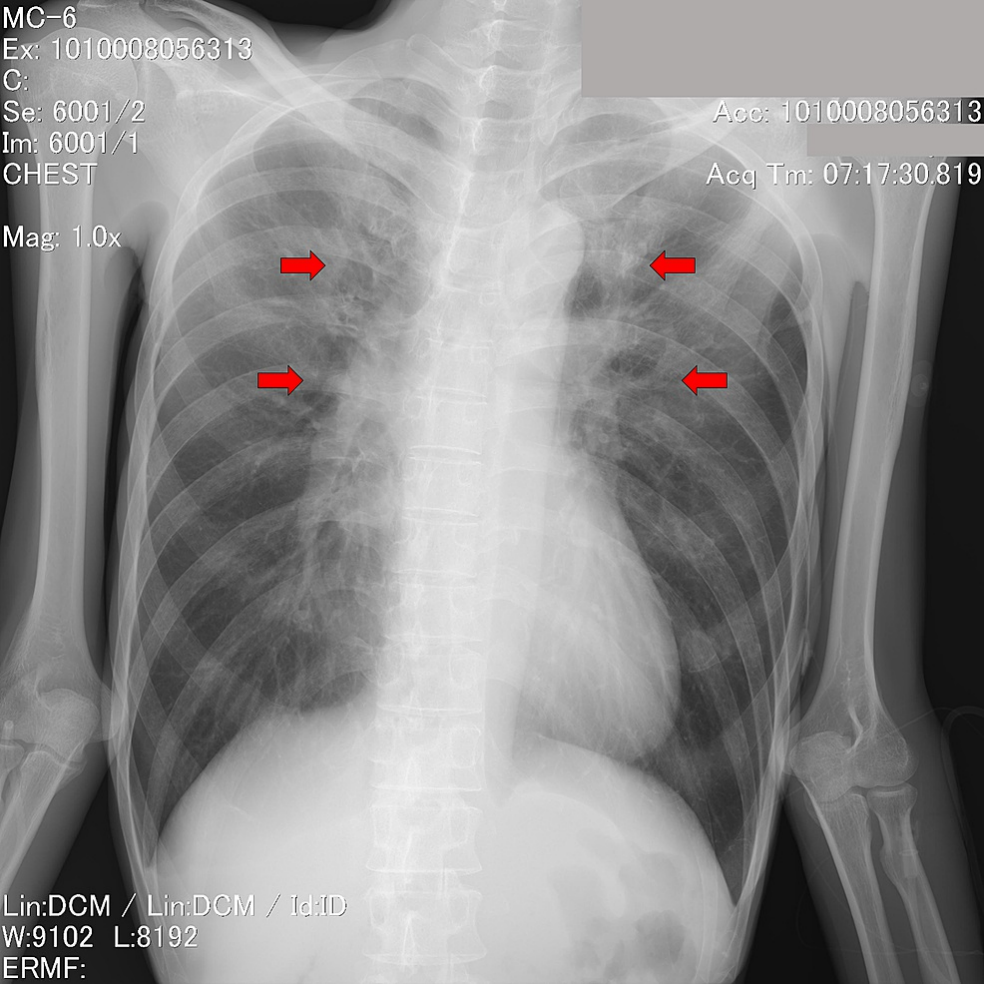
Chest X-ray at admission Initial chest X-ray demonstrated mild interstitial pulmonary edema (red arrows).

Initial chest X-ray demonstrated mild interstitial pulmonary edema (Figure 2). 

**Figure 3:**
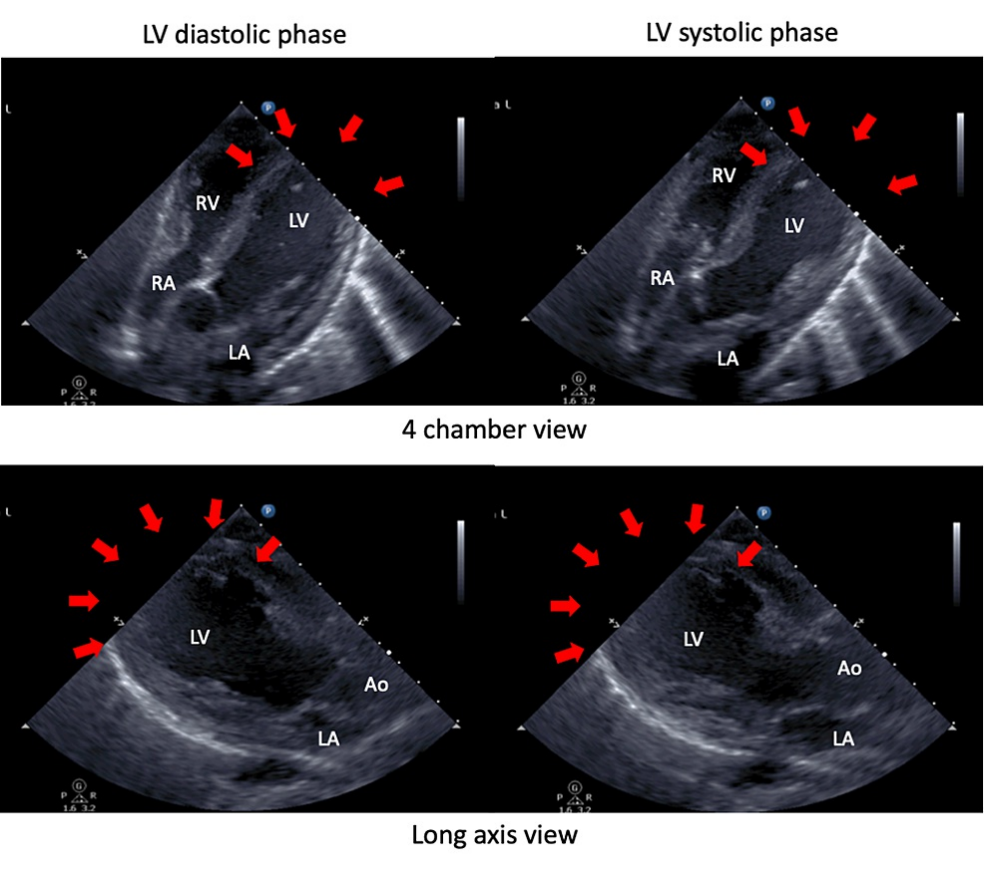
Two-dimensional echocardiogram at admission Echocardiography revealed a severely decreased LV ejection fraction of 20%, with global hypokinesis and apical akinesia (red arrows). End-diastolic (A) and end-systolic (B) frames at admission showed dyskinesia of apex and middle segment of the LV. Ao, aorta; LA, left atrium; LV, left ventricle; RA, right atrium; RV, right ventricle.

Echocardiography revealed a severely decreased LV ejection fraction of 20%, with global hypokinesis and apical akinesia (Figure 3, Video 1 and Video 2).

**Video 1:** Apical four-chamber echocardiogram view at admission Apical four-chamber echocardiogram view reveals a severely decreased left ventricular ejection fraction of 20%, with global hypokinesis and apical akinesia.

**Video 2:** Parasternal long-axis echocardiogram view at admission Parasternal long-axis echocardiogram view reveals a severely decreased left ventricular ejection fraction of 20%, with global hypokinesis and apical akinesia.

**Figure 4:**
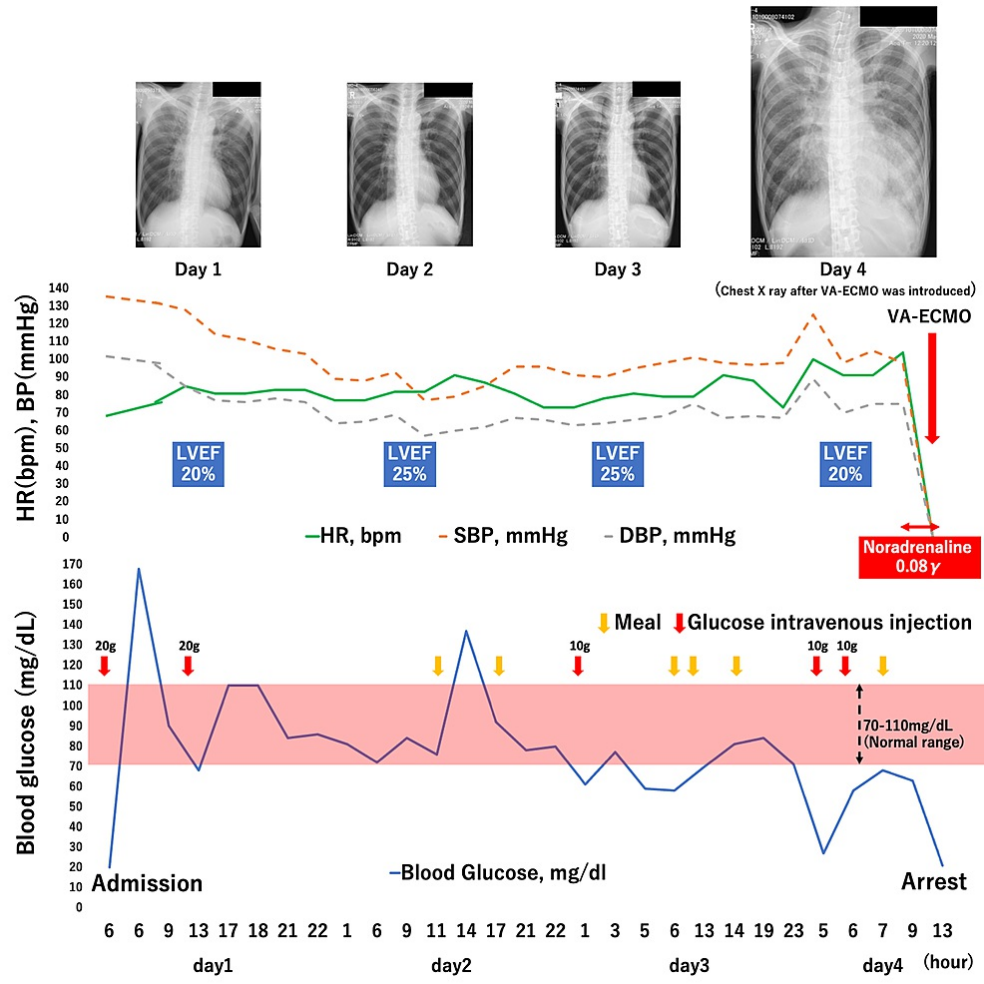
The patient’s clinical course and blood glucose trends bpm, beats per minute; DBP, diastolic blood pressure; HR, heart rate; LVEF, left ventricular ejection fraction; SBP, systolic blood pressure; VA-ECMO, venoarterial extracorporeal membrane oxygenation.

He had no coronary risk factors. ECG revealed no significant ST change and echocardiography revealed LV apical ballooning that indicated TC. From these findings, we speculated that starvation and subsequent intractable hypoglycemia had caused TC. Although he did not have the history of alcohol use disorder, chronic viral hepatitis, or other liver disease, the patient showed the marked elevations in AST and ALT. Due to poverty and schizophrenia-related symptoms, the patient’s daily food intake was insufficient, and he developed severe weight loss. We, therefore, confirmed that a possible cause of liver failure may be starvation. Ultrasonography demonstrated normal in size and normal echogenicity, indicating starvation-related liver enzyme elevation [7]. We planned to slowly increase his nutritional intake according to the National Institute for Health and Clinical Excellence (NICE) guidelines, while managing refeeding syndrome (RFS) [8]. However, the hypoglycemia was refractory and recurrent, requiring repetitive glucose administration (Figure 4).

Thiamine, phosphorus, magnesium, and potassium levels were also strictly monitored and supplemented appropriately (Table 1).

**Table 1:** The patient’s clinical course and nutrition and electrolyte replenishment AST, aspartate aminotransferase; ALT, alanine aminotransferase; CK, creatine kinase; CRP, C-reactive protein; K, kalium (potassium); Mg, magnesium; Na, natrium; P, phosphorus; PT-INR; prothrombin time-international normalized ratio.

		Day 1				Day 2			Day 3		Day 4	
Hour		6	13	18	22	6	14	22	6	14	6	14
Complete blood count												
White blood cell (/μL)	3300-8600/μL	3700		7500	7200	8090			8910		4030	1300
Neutrophils (%)	38.5-80.5%					87.1			80.9		88.3	80.4
Lymphocytes (%)	16.5-49.5%					8.5			15.3		11.0	18.7
Hemoglobin (g/dL)	13.7-16.8 g/dL	9.9		13.1	13.3	13.4			13.9		14.4	4.8
Hematocrit (%)	40.7-50.1%	30.3		39.2	39.7	40.6			42.3		43.8	14.7
Platelet x10^4 ^(/μL)	15.8-34.8 x 10^4^/μL	77		102	96	92			79		56	8
PT (INR)	0.90-1.15	2.60	1.89		2.25	2.21			1.93		2.40	>10.00
Biochemical data	Reference range											
Troponin I (pg/mL)	<0.040 pg/mL		0.313	1.291	1.659	1.121	0.672		0.382	0.324	0.217	10.364
CK (IU/L)	59-248 IU/L	405		659	709	718	721		618	656	593	198
AST (U/L)	13-30 U/L	870		880	823	676			495		1623	263
ALT (U/L)	13-30 U/L	682		854	822	748			631		1159	213
Na (mEq/L)	138-145 mEq/L	134	135	135	134	134	133	129	130	130	130	130
K (mEq/L)	3.6-4.8 mEq/L	3.9	4.1	4.1	3.9	4.1	4.2	4.2	4.3	4.3	4.1	7.4
P (mEq/L)	2.7-4.6 mEq/L	5.3	4.2	3.5	3.5	3.3	3.4	2.9	3.1	3.4	3.8	6.1
Mg (mEq/L)	2.0-2.4 mEq/L	3.0	2.4	2.3	2.3	2.3	2.3	2.2	2.1	2.1	2.1	1.8
CRP	0.000-0.014 mg/dL	<0.015				0.667			0.364		0.821	0.714
Lactate (mmol/L)	0.5-1.6 mmol/L	1.3	0.9		1	0.6	1.5	0.6	0.5	0.7	1.1	7.8
Nutrition and electrolyte replenishment												
Parenteral nutrition (kcal)		300				450			600		300	
Enteral nutrition (kcal)		0				200			200		300	

He was treated with IV thiamine 500 mg three times a day. His phosphorus, magnesium, and potassium levels were sufficiently maintained by meal content and maintenance infusion. Therefore, we did not add oral or parenteral supplementation. On day 2, the ECG showed a negative T wave anteriorly, indicating typical ECG changes in TC, but excessive QT prolongation was not noted (QTc: 480 ms) (Figure 1B). Although echocardiography revealed no remarkable recovery of the LV wall motion, congestive heart failure improved without using any diuretics, and he had an oxygen saturation of ≥95% on room air after admission. His hemodynamic parameters including blood pressure, heart rate, and lactate maintained stability and he did not need any catecholamines support until day 4 (Figure 4). On day 4, the patient developed severe refractory hypoglycemia and required repetitive glucose infusion. He became drowsy and his systolic blood pressure progressively decreased. Subsequently, pulseless ventricular tachycardia followed by pulseless electrical activity developed. Return of spontaneous circulation could not be achieved by conventional cardiopulmonary resuscitation, and venoarterial extracorporeal membrane oxygenation (VA-ECMO) was introduced. However, the VA-ECMO could not be maintained, and the patient died on the fourth day of admission.

**Figure 5:**
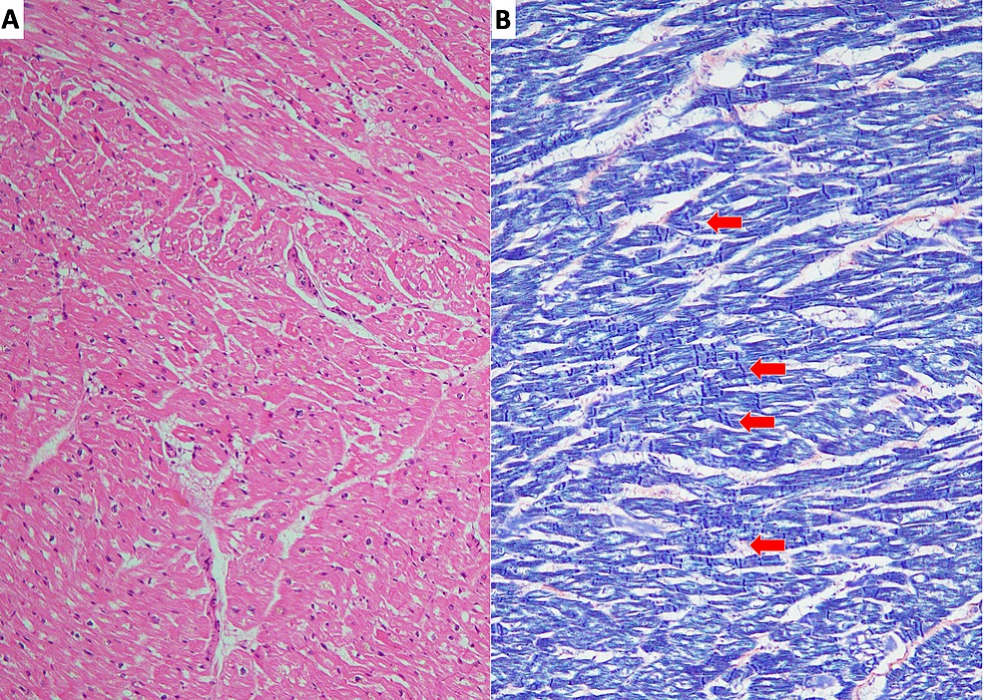
Microscopic view of heart (A, B). A low-power view of cardiac muscle showed diffuse myocardial atrophy and degeneration. (H&E staining) (A). Contraction band necrosis was observed (red arrows). (Phosphotungstic acid hematoxylin staining) (B). H&E, hematoxylin and eosin stain.

On autopsy, myocardial atrophy, degeneration, and contraction band necrosis were widely noted in both ventricles, regardless of the distribution of coronary arterial branches, consistent with TC (Figure 5A, 5B).

**Figure 6:**
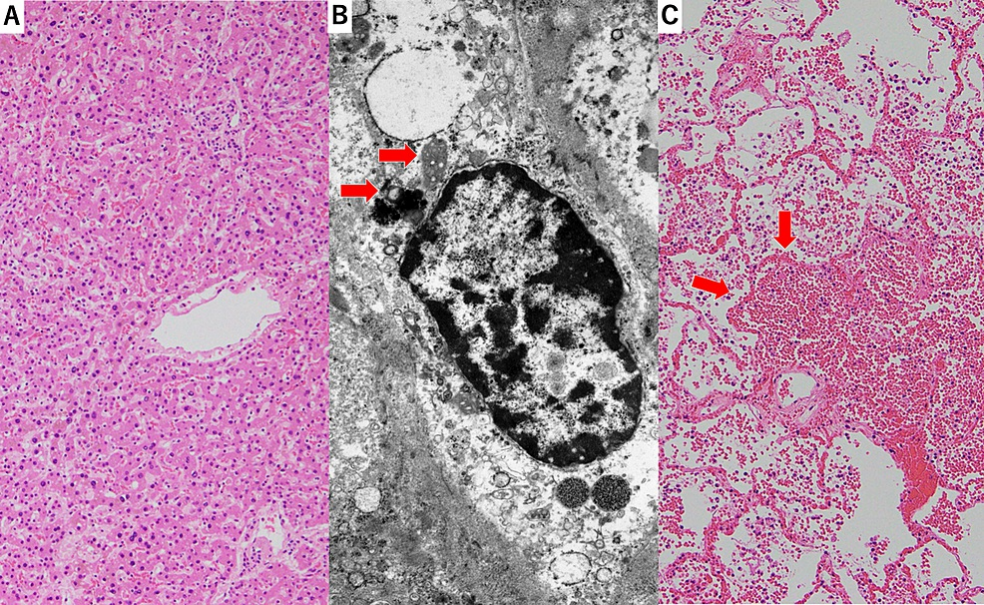
Microscopic view of the liver (A, B) and lung (C) A low-power view of the liver showed diffuse hepatocyte atrophy. (H&E staining) (A). A view of hepatocytes observed by electron microscopy showed autophagosome (red arrows) and perinuclear space (B). A low-power view of lung showed blood accumulation in the alveolar spaces (red arrows). (H&E staining) (C). H&E, hematoxylin and eosin stain.

Coronary artery stenosis and myocardial infarction were not observed. The liver showed moderate atrophy, extensive iron deposition, as well as autophagosomes and perinuclear spaces, which are features of autophagic cell death. These findings are consistent with liver injury due to starvation, but not with RFS (Figure 6A, 6B) [9].

Mucosal hemorrhage extending from the trachea to the bronchi of all lobes of the lungs and accumulation of blood in the alveolar spaces was also observed (Figure 6C). Coagulation abnormalities due to liver dysfunction are thought to have been associated with severe bleeding. We finally diagnosed that the patient suffered fatal ventricular tachyarrhythmia and extremely low cardiac output induced by TC, and VA-ECMO could not be maintained due to massive lower respiratory bleeding caused by chest compression.

## Discussion

The prognostic impact of refractory hypoglycemia in the patients with TC with starvation has not been fully elucidated. However, several case reports indicated that refractory hypoglycemia is associated with cardiac complications, such as cardiogenic shock, low cardiac output, and fatal arrhythmia in patients with starvation complicated by TC [5,6,10]. Patients in the terminal stages of starvation, when gluconeogenesis from proteins is no longer possible, are likely to have refractory hypoglycemia [6]. Additionally, endogenous glucose production and fatty acid oxidation are reduced when glucose is administered or the patient is fed after a long period of starvation, which may result in the excessive secretion of insulin to maintain euglycemia [6,11]. These mechanisms result in refractory hypoglycemia in patients with terminal-stage starvation. As a result, refractory hypoglycemia causes markedly elevated catecholamine levels, which are associated with the onset and deterioration of TC [12]. Furthermore, endogenous secretion of too much insulin induced by rapid intravenous glucose injection during the treatment of hypoglycemia due to malnutrition was also reported to be associated with fatal arrhythmia [5]. Secretion of too much insulin activates the sodium-potassium-adenosine triphosphate pump, which extrudes sodium from cells in exchange for potassium [12]. Increased intracellular potassium inactivates delayed rectifier K channel of phase 3 in the action potentials, and action potential duration is prolonged. Accordingly, the QT interval can be prolonged, leading to ventricular arrhythmia [5]. Based on these findings, we thought that patients with TC accompanied by starvation with refractory hypoglycemia who require repetitive glucose injections can develop extreme cardiac output depression and fatal ventricular arrhythmia, resulting in a poor prognosis. Patients with hypoglycemia and starvation should undergo repeated cardiovascular examinations with ultrasound, electrocardiogram, chest X-ray, and cardiac enzymes for early recognition of new onset and complications of TC [13].　

RFS also induces severe cardiac complications, such as heart failure and ventricular tachyarrhythmia [10]. However, the present case was well recognized as being at a high risk for RFS; therefore, calorie uptake was increased carefully according to the NICE guidelines [14]. In fact, the liver autopsy showed the findings of autophagic cell death, consistent with starvation and not RFS. Autophagy, a lysosomal catabolic pathway for long-lived proteins and damaged organelles, is crucial for cell homeostasis, and survival under stressful conditions. Autophagy provides amino acids and participates in glucose metabolism following starvation. In patients with starvation, autophagy appears initially protective, allowing cells to cope with nutrient deprivation. However, when starvation is critically prolonged, acute liver insufficiency occurs with features of autophagic cell death, which can be observed by electron microscopy analysis of liver biopsy samples [15], as presented in the present case. The histological findings of the liver in RFS were hepatic fat and glucose deposition, known as steatosis. These findings were not observed in the present case [16,17].

The NICE guidelines recommend that energy input should start at a maximum of 5 kcal/kg daily and increase slowly to reach the maximum in 4-7 days to avoid RFS in extreme cases of starvation in a person with body mass index <14 kg/m^2^ [8]. However, a previous report indicated that patients with refractory hypoglycemia during starvation need two to five times more calories than that recommended by NICE to maintain euglycemia [6]. Additionally, a recent study reported that real-time continuous glucose monitoring in critically ill patients offers unique benefits by allowing real-time tracking of glucose levels to detect often-unrecognized severe hypo- or hyperglycemic events [18]. Adequate caloric intake and stricter blood glucose control with continuous real-time glucose monitoring may be useful for timely intervention to prevent excessive caloric administration and severe hypoglycemic events and improve outcomes. Further studies are needed to determine how to identify and treat patients with cardiac complications related to hypoglycemia and starvation.

## Conclusions

We treated a patient with TC accompanied by starvation with refractory hypoglycemia who did not survive due to cardiac complication of TC. To our knowledge, this is the first report of TC secondary to hypoglycemia and starvation in which the cause of death was confirmed by autopsy. We speculated that refractory hypoglycemia accompanied by starvation induced extreme catecholamine secretion, which led to severe complications of TC, such as fatal arrhythmia and extremely low cardiac output. We recommend that frequent glycemic monitoring, strict glycemic control, and adequate caloric intake should be ensured for patient with TC secondary to hypoglycemia and starvation. Moreover, to avoid severe cardiac complications, close cardiac monitoring should be done with ECG, serial troponins, and an echocardiography. Further clinical trials would be needed to determine how to identify and treat patients with cardiac complications related to hypoglycemia and starvation.
